# Development of an Advanced Dynamic Microindentation System to Determine Local Viscoelastic Properties of Polymers

**DOI:** 10.3390/polym11050833

**Published:** 2019-05-08

**Authors:** Esther Ramakers-van Dorp, Thomas Haenel, Dominik Ciongwa, Bernhard Möginger, Berenika Hausnerova

**Affiliations:** 1 Department of Natural Sciences, University of Applied Sciences Bonn-Rhein-Sieg, von Liebigstrasse 20, 53359 Rheinbach, Germany; esther.vandorp@h-brs.de (E.R.-v.D.); dominik.ciongwa@smail.bcw.h-brs.de (D.C.); bernhard.moeginger@h-brs.de (B.M.); 2 Faculty of Technology, Tomas Bata University in Zlín, Vavrečkova 275, 760 01 Zlín, Czech Republic; 3 Netzsch Gerätebau GmbH, Wittelbachstrasse 42, D-95100 Selb, Germany; thomas.haenel@netzsch.com; 4 Centre of Polymer Systems, Tomas Bata University in Zlín, tr. T.Bati 5678, 760 01 Zlín, Czech Republic

**Keywords:** dynamic indentation, dynamic mechanical analysis, tungsten cone indenter, complex modulus, spatial resolution

## Abstract

This study presents a microindentation system which allows spatially resolved local as well as bulk viscoelastic material information to be obtained within one instrument. The microindentation method was merged with dynamic mechanical analysis (DMA) for a tungsten cone indenter. Three tungsten cone indenters were investigated: tungsten electrode, tungsten electrode + 2% lanthanum, and tungsten electrode + rare earth elements. Only the tungsten electrode + 2% lanthanum indenter showed the sinusoidal response, and its geometry remained unaffected by the repeated indentations. Complex moduli obtained from dynamic microindentation for high-density polyethylene, polybutylene terephthalate, polycarbonate, and thermoplastic polyurethane are in agreement with the literature. Additionally, by implementing a specially developed x-y-stage, this study showed that dynamic microindentation with a tungsten cone indenter was an adequate method to determine spatially resolved local viscoelastic surface properties.

## 1. Introduction

Micro- and nanoindentation and dynamic mechanical analysis (DMA) are methods used to determine mechanical and viscoelastic material properties. Almost all materials show a local distribution of their properties reflecting their heterogeneous structure or non-uniform processing conditions. Therefore, materials respond to deformation differently on a local or bulk scale. The deformation properties can be distinguished as mechanical (such as hardness) and as viscoelastic (such as storage and loss moduli) properties. Instrumented micro- and nanoindentation provide information about, for example, material stiffness, indentation hardness, and indentation modulus by evaluating loading and unloading curves during loading and unloading, respectively. Oliver and Pharr [1,2] made an enormous contribution to the development and the analysis of the indentation technique. Based on the theories of Hertz [3] and Sneddon [4], Oliver and Pharr developed the evaluation for several indenter geometries, for example, for the three-sided diamond Berkovich indenter, which is now commonly used (see Theoretical Consideration).

Polymers are complex viscoelastic materials which are strongly time- and temperature- dependent. Viscoelastic behavior is accessible with time- and/or frequency-dependent measurements. The dynamic mode of the instrumented indentation, imposing a small sinusoidal deformation, allows the determination of local viscoelastic properties on a small scale. The sinusoidal response signal can be separated using the Euler’s equation into a real part and an imaginary part representing storage and loss moduli, respectively. The storage modulus is a measure of the elastic response of a material behavior, whereas the loss modulus reflects the viscous material behavior. The quotient of the loss and storage moduli is the loss factor which represents the material damping behavior. The attractiveness of this measuring technique is the determination of viscoelastic properties within one measurement. The DMA is the original and standard measuring technique which uses the sinusoidal deformation to determine viscoelastic properties.In a previous work [5], we presented a new quasi-static and dynamic microindentation method by merging indentation with conventional DMA to broaden the capability of DMA. It was shown that for standard diamond indenters according to Berkovich, Vickers, and Rockwell, the quasi-static and dynamic microindentations were successfully performed. This study demonstrated that microindentation with a conventional DMA allows for the determination of not only bulk viscoelastic properties, but also of local viscoelastic properties within one instrument [5].

However, the previous work [5] showed that for a tungsten cone indenter, some issues occurred: (i) the resonant frequency spectra showed overtones >10% of the first order of the resonant frequency (i.e., outside manufacturer specifications), (ii) the raw response signal did not show a sinusoidal signal, (iii) the indenter geometry was affected (bent/broke) after a few indentations, (iv) the precise, spatially resolved measurements were impossible to obtain, and (v) the spatial resolution was limited in contrast to tungsten cone indenters due to the large surface area of a standard diamond indenter. Therefore, the solution for the above-mentioned problems is addressed in this work, together with general disadvantages reported about micro- and nanoindentation, such as instrument frame compliance, influence of surface energies, and indenter tip imperfections. This was done by investigating different tungsten cone indenter materials with a larger diameter as in the previous study [5]. Furthermore, a specially developed x-y-stage was implemented which enabled spatially resolved microindentations. Solving these problems finalizes the newly developed DMA-microindentation method, offering the possibility to determine not only the bulk viscoelastic material properties but also the local viscoelastic material properties within the conventional DMA.

## 2. Theoretical Consideration

Instrumented indentation allows for measuring the stiffness given by the slope of the linear section at the beginning of the unloading curve. Oliver and Pharr [1,2] developed a method to extract Young’s modulus using stiffness *S*. The contact stiffness *S* of the sample is related to the reduced modulus *E_r_* and the indenter contact area *A*:

(1) $$S={\left(\frac{dP}{dh}\right)}_{h=h_{max}}^{unload}=\frac{2}{\sqrt{\pi }}*E_{r}*\sqrt{A}.$$

The reduced modulus *E_r_* depends on moduli and Poisson’s ratios of indenter (*E_i_*, *ν_i_*) and sample (*E_s_, ν_s_*), respectively:

(2) $$\frac{1}{E_{r}}=\frac{1-\nu_{s}^{2}}{E_{s}}+\frac{1-\nu_{i}^{2}}{E_{i}}\;\Leftrightarrow \;E_{r}=\;\frac{E_{s}\;E_{i}}{E_{i}\;\left(1-\nu_{s}^{2}\right)+E_{s}\;\left(1-\nu_{i}^{2}\right)\;}.$$

Merging Equations (1) and (2) yields the following:

(3) $$S=\frac{2}{\sqrt{\pi }}\times \frac{E_{s}\;E_{i}}{E_{i}\;\left(1-\nu_{s}^{2}\right)+E_{s}\;\left(1-\nu_{i}^{2}\right)\;}\times \sqrt{A},$$

and resolving with respect to the Young’s modulus of the sample *E_s_*:

(4) $$E_{s}=\frac{\frac{\left(1-\nu_{s}^{2}\right)}{2}\times \frac{\sqrt{\pi }}{\sqrt{A}}\times S}{1+\frac{\left(1-\nu_{i}^{2}\right)}{2\times E_{i}}\times \frac{\sqrt{\pi }}{\sqrt{A}}\times S}.$$

Since an indenter material is much stiffer than a polymer sample (*E_i_* >> *E_s_*), the second term in the denominator is a small quantity and can be neglected [1,6,7,8,9]:

(5) $$E_{s}=\frac{\left(1-\nu_{s}^{2}\right)}{2}\times \frac{\sqrt{\pi }}{\sqrt{A}}\times S=\frac{\left(1-\nu_{s}^{2}\right)}{2}\times \frac{\sqrt{\pi }}{\sqrt{A}}\times \frac{dP}{dh}.$$

From the dynamic indentation, the ratio of the measured complex load amplitude $F_{s}^{*}$ and the complex displacement amplitude $A_{s}^{*}$ can be taken for the ratio *dP/dh*, Equation (5). Consequently, the complex modulus E* can be expressed as follows:

(6) $$E^{*}=\frac{F_{s}^{*}}{A_{s}^{*}}\times \frac{\left(1-\upsilon_{s}^{2}\right)}{2}\times \frac{\sqrt{\pi }}{\sqrt{A}}.$$

Equation (6) depends on the indenter geometry by the contact area *A*, whereby the contact area *A* is a function of the displacement *h*. For the tungsten cone indenter, this can be easily calculated using the trigonometrical function for a cone:

(7) $$A=\pi \times tan^{2}\alpha \times h^{2},$$

where *α* is the half opening angle of the cone indenter.

Important issues that generally have to be considered during indentation are (a) instrument frame compliance, (b) determination of first contact between indenter and material surface, (c) surface energies such as adhesive or compulsory energies between indenter and material surface, (d) indentation size effects, (e) tip geometry imperfections, and (f) piling up or sinking in of a material.

Exact knowledge of the instrument frame compliance is essential to obtain exact results [7,10]. During indentation, a combined stiffness is actually measured, which consists of the stiffness of the instrument and of the sample. An accurate calibration of the instrument frame compliance ensures that only the sample response from the indentation is evaluated, that is, any possible influence of the instrument on the measurement can be excluded. The first contact of the indenter with the sample surface is essential for an adequate evaluation of the results. The first contact can be determined by monitoring force and indentation at a high indenter speed, and the point of contact is set where the force shows a sharp rise [11]. Alternatively, the first contact is considered if the contact force exceeds a preset small value that is setting a depth sensor to zero. Afterwards, the force indentation is fitted to a curve, and then extrapolated to a minimum. This correction has the effect of shifting curves to higher loads and displacements [12].

Instrumented nanoindentation works with lower loads and displacements, thus the effect of surface energies and related adhesive forces may become relevant or even dominate the overall force behavior on a nanoscale. The Johnson–Kendall–Roberts (JKR) theory is applicable for characterizing contacts of compliant samples with high surface energies, that is, strong adhesive forces between sample and indenter. The Derjaguin–Muller–Toporov (DMT) theory is more suitable for stiffer samples, low but not negligible surface energies, and probed by a sharp indenter. A theory for the intermediate regime is covered by Maugis–Dugdale (MD) [11,13,14,15]. Since this study works on a microscale with relatively high loads and large displacements, the surface energy effect will be neglected.

The indentation size effect (ISE), a scale-dependent behavior as the hardness increases with decreasing penetration depths, may become a relevant effect at penetration depths lower than 1 µm or for spherical indenters with radii less than 100 µm [16]. An apparent ISE can be caused by sample surface contaminations, mechanical damage due to a surface preparation, and surface roughness. Furthermore, improper indenter area–function calibration, indenter tip rounding or blunting, or errors in instrument compliance calibration can simulate the presence of ISE [16]. In comparison with studies about metals, the literature on the ISE of polymers is scarce and the mechanism is not well understood [17,18,19]. Since this study works with displacements larger than 1 µm, the ISE will not be considered.

Instrumented indentation analysis is based on an elastic contact theory assuming that sinking-in, when a material is pulled down toward an indenter tip, occurs in the indentation region. However, if piling-up (material is pushed upward along the indenter), the contact depth exceeds the measured indentation depth used for analysis, and the calculated elastic modulus becomes overestimated, as seen in Equations (6) and (7) [20,21,22]. Unlike studies about other materials, discussion concerning piling-up and sinking-in for polymers is rather contradictory [20,23,24,25]. Since the effect of piling-up on the overestimation of the calculated elastic modulus becomes smaller at high indentation depths, and there is still no standard procedure to determine the correct contact area [22], piling-up or sinking-in will be disregarded in this study as well [26].

The evaluation of the indentation results according to the Oliver and Pharr method is based on concise axisymmetric indenter geometry. In reality, this is hardly realizable, and for small depths the shape at the end of the tip is critical to obtain meaningful results. The standard procedure for micro- and nanoindentation to deal with indenter geometry imperfections is to carry out so-called tip calibration, by calibrating the indenter area function on two different materials with uniform and well-known material properties (e.g., sapphire and fused quartz) [6,11]. Corrections obtained from the tip calibration are then used for further computational data evaluation. As the software of the conventional DMA (also used in this study) does not allow for a computational correction, the indenter geometry has to be evaluated optically with a calibrated light microscope. If the indenter is not ideal and tip bluntness is present, the contact depth can be modified according to Troyon and Huang [20] by relating the geometry defect to the indenter contact area, which is sufficiently consistent for indenter displacements higher than 200 nm:

(8) $$A=\pi \times tan^{2}\alpha \times {\left(h+h_{b}\right)}^{2},$$

where *h_b_* is the truncation length of the indenter tip defect.

## 3. Materials and Methods

### 3.1. Materials

**Figure 1:**
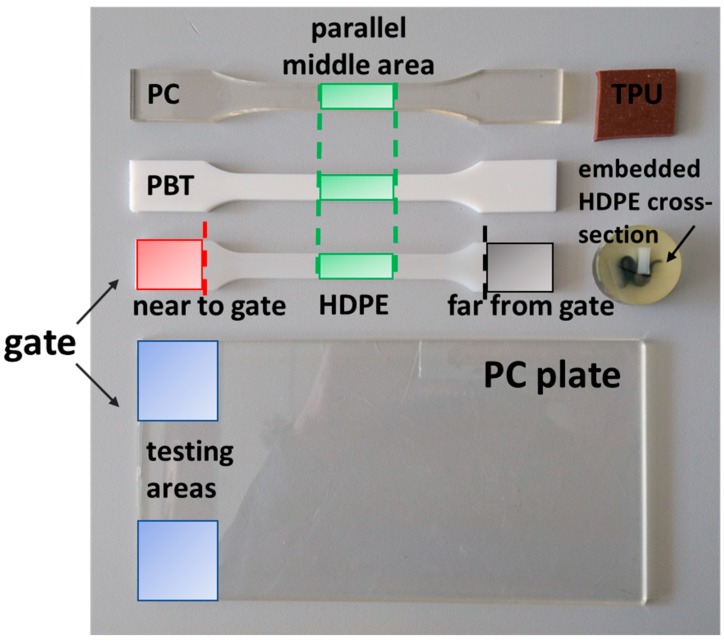
Photograph of sample positions for the different materials.

Four types of polymers were tested, as is shown in Table 1. High-density polyethylene (HDPE), polybutylene terephthalate (PBT), and polycarbonate (PC) injection molded tensile test bars (ISO 527-2:1A) were investigated in the narrow parallel area in the middle of the bar (see Figure 1). HDPE is a semi-crystalline polymer with glass transition temperature (*T*_g_) below room temperature, PBT is a semi-crystalline polymer with *T*_g_ above room temperature, PC represents an amorphous polymer with *T*_g_ above room temperature, and thermoplastic polyurethane (TPU) is an elastomeric polymer with *T*_g_ below room temperature. All were chosen to show a broad range of thermo-mechanical properties.

In order to enhance the measurement reliability, a neat injection molded PC plate (3 × 100 × 250 mm), which was specially produced for the adhesion testing of coatings (UL, Underwriters Laboratories, Cologne, Germany), was tested as well. From this plate, two samples (30 × 30 mm) were carefully cut near to the gate, as is shown in Figure 1. One of these samples was further annealed for 2 h at 110 °C to consider the possible influence of sample processing. Additionally, HDPE samples were investigated at the positions corresponding to the gate-far and gate-near region (see Figure 1), before and after annealing at 90 °C for 2 h. TPU samples were punched out of a pattern pad.

The dynamic microindentations were performed with three different tungsten consumable welding electrodes (Litty, Grabenstaett Germany) as cone indenters (see Table 2).

### 3.2. Apparatus

**Figure 2:**
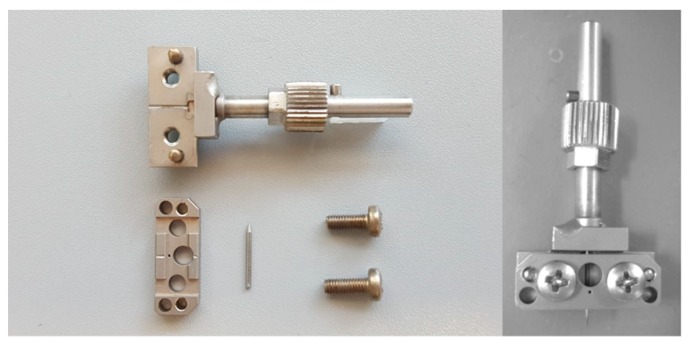
Indenter holder for cone indenter.

A DMA (242 C, Netzsch, Selb, Germany) was used to perform dynamic microindentations on selected polymers. For the microindentations, a special DMA indenter holder was used for the cone indenter, as is shown in Figure 2. Aligned to the probe rod, the cone indenter was placed into the notch of the sample holder. Then the sample holder was covered with the notched sample holder lid and fixed into place with two screws. Finally, the indenter holder was implemented into the DMA.

An oscilloscope (TPS2012B, Tektronix, Beaverton, OR, USA) was connected to the DMA to collect raw data of the time-dependent force and deformation during the dynamic microindentation. Fast Fourier transformation (FFT) of the raw data was used to evaluate the accuracy of the dynamic microindentations. A divergence in the sinusoidal form of the response signal results in a higher order of resonant frequencies, which are tolerable within less than 10% of the first order resonant frequency.

**Figure 3:**
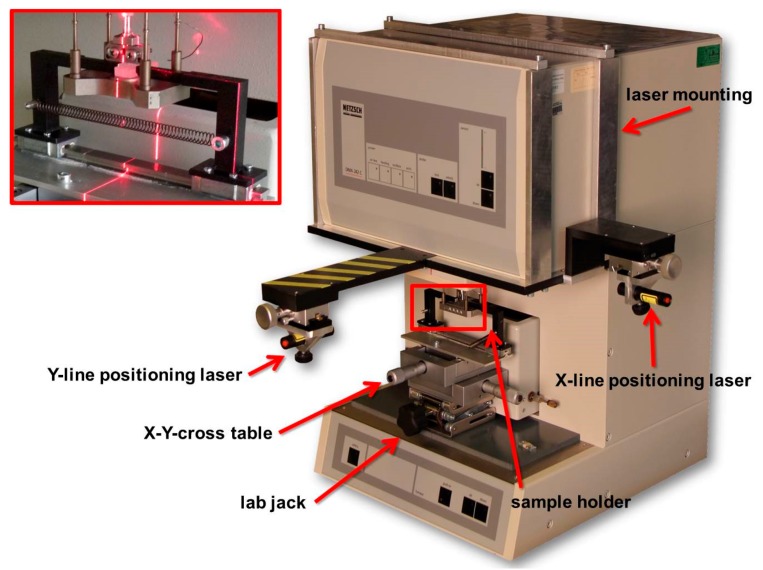
Specially developed x-y-stage with a laser positioning system for spatial resolution of microindentations.

To perform spatially resolved measurements, a special x-y-stage with a laser positioning system was developed and adapted to the DMA (see Figure 3). With the lab jack and the x-y-cross table, the sample can be positioned easily. The sample was fixed onto the sample holder between two angled hooks, which were tightened by a tension spring. The reflection of the x- and y-positioning laser showed the exact location on the sample, where the indenter would penetrate. This gives an optimal overview and makes specimen adjusting easier. Spatially resolved measurements can be obtained by turning the x- and/or y-screws with a resolution of 1 µm between indentations.

### 3.3. Measurements

The dynamic microindentations were performed in the standard penetration mode with the different tungsten cone indenters to determine the complex modulus E* and the resonant frequency spectra. The DMA 242C was calibrated with a 3 mm flat punch in accordance with the procedures provided by Netzsch. The measurements were performed at room temperature and at a frequency of 1 Hz, and consisted of two segments. The first segment was displacement controlled and consisted of a maximum deformation amplitude of 0.10 µm and a maximum load of 0.5 N to ensure contact of the indenter with the sample surface and to set a zero position for the following displacement measurement. The second segment started after reaching the equilibrium state of the first segment. It was a force-controlled segment with a maximum deformation amplitude of 120 µm and loads ranging from 0.5 N up to 1.25 N to investigate the influence of the load on the resonant frequency spectra. For both segments, 10% of the maximum load was used as a constant static load (proportionality factor 1.1) to ensure that the indenter would not lift off from the sample during the measurement [5]. Each dynamic microindentation was repeated five times.

The complex load amplitude $F_{s}^{*}$ and the complex displacement amplitude $A_{s}^{*}$ were taken at the end of the second segment, where the maximum force was reached, and the amplitude as well as the penetration depth reached equilibrium. The data were used to calculate the complex modulus E*. In addition, at the end of the second segment, the plastic deformation was expected to be completed, and the penetration depth *dh* was used to calculate the indentation area *A*.

Each tungsten cone indenter was checked with light microscopy to confirm axisymmetric geometry with a sharp tip and a tip angle of 28° before implementing it into the DMA 242C and after each set of five dynamic microindentations.

## 4. Results and Discussion

In order to investigate optimal testing conditions for tungsten cone indenters, load, and overtones of the first order frequency, dynamic microindentations were performed on the narrow parallel area in the middle of the injection molded HDPE tensile bars. In the earlier study [5], we found that for a tungsten cone indenter with a diameter of 0.5 mm, the overtones of the first order resonant frequency were considerably higher (55%) than 10% proposed by the manufacturer of the DMA 242C. For this study, we decided to use tungsten cone indenters with a diameter of 1 mm, because the larger diameter of the cone indenter should result in a stiffer and more stable measuring system, thereby reducing the overtones of the first order resonant frequency.

From at least five measurements per experimental combination, the mean values of the overtones of the first order frequency and the complex modulus were determined for HDPE (see Table 3). As can be seen in Table 3, all tungsten cone indenters showed overtones less than 10% of the first order resonant frequency under loads ranging from 0.5 N to 1.25 N, which corresponds to the manufacturer’s specifications for the DMA 242C instrument. Thus, changing the indenter diameter from 0.5 mm to 1 mm resulted in a substantial decrease in the overtones of the first order resonant frequency. Furthermore, Table 3 shows the calculated complex moduli from the dynamic microindentations for various tungsten cone indenters. If compared with the material datasheet [30], where the Young’s modulus of Lupolen 4261AG is reported to be 900 N/mm^2^, all measurements show higher values at different loads. This discrepancy might result from the fact that the Young’s modulus from the material datasheet represents the mean value of a bulk property from tensile testing, whereas the values obtained in this study represent a local surface property.

Local properties are clearly more sensitive to local structure differences (e.g., inhomogeneities). For the tested load variations, the measurements at 1.25 N showed the complex moduli with the lowest standard deviation (average of five measurements) for all three tungsten cone indenters.

**Figure 4:**
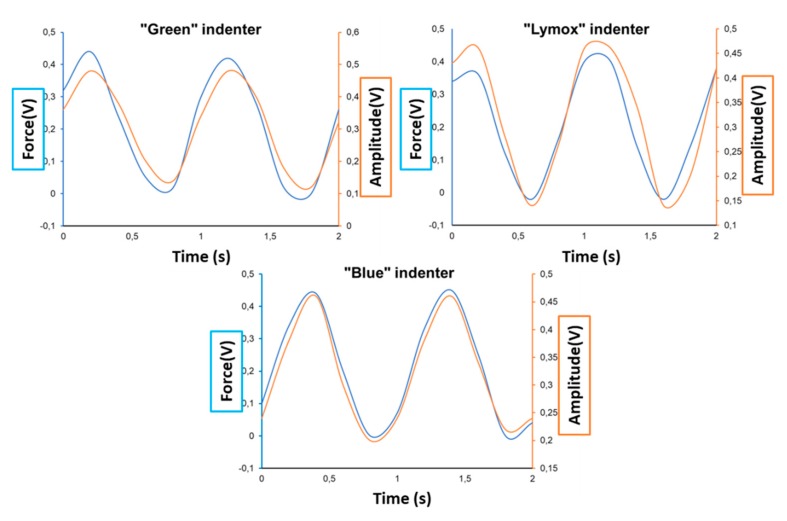
Evolution of raw applied signal force and raw response signal amplitude (both processed with fast Fourier transformation (FFT)) for various tungsten cone indenters.

Figure 4 shows the raw applied force signal and the raw response signal amplitude, both in volts, for all three different tungsten cone indenters to evaluate the sinusoidal response signal. In Figure 4, it can be seen that the tungsten electrode (Green) and the tungsten electrode with rare earth elements (Lymox) gave no clear sinusoidal response signal, and only the tungsten electrode with 2% lanthanum (Blue) indenter responded with a smooth sinusoidal signal.

An additional reason to choose the tungsten electrode with 2% lanthanum indenter for further measurements were the shortcomings resulting from dynamic microindentation such as bending, breaking, and/or splintering off the indenter tip. For all three different tungsten cone indenters, the indenter holder was removed from the DMA 242C after each set of five indentations and the indenter tip was checked under a light microscope. The Green and the Lymox indenters were affected after 5 to 10 and 20 to 25 indentations, respectively, but the Blue indenter was still not affected after 250 indentations.

The subsequent dynamic microindentations were performed with the tungsten electrode with 2% lanthanum (Blue) indenter upon load of 1.25 N. The results for the complex moduli of the four types of polymers, determined by dynamic microindentations with the tungsten cone indenter, dynamic microindentations with different diamond indenters, three-point bending with the DMA 242 C, both from the earlier study [5], and Young’s moduli from literature [27,28] are listed in Table 4. The comparison of the complex moduli of PBT, PC, HDPE and TPU measured by dynamic microindentation with the tungsten cone indenter shows a good agreement with the three-point bending complex moduli and literature values. Since the three-point bending moduli and literature values, which originate from tensile testing, represent bulk mechanical properties, some deviation between the literature values and the local viscoelastic complex moduli can be expected, as mentioned above.

A noticeable observation is that the complex moduli determined with the tungsten cone indenter for PBT and PC show the highest values with a relatively high standard deviation, but slightly above the maximum of the literature [27,28] values. To explain this deviation, the reproducibility of the tungsten cone indenter was evaluated on a neat injection molded PC plate. Neat PC was chosen for this part of the study, because nanoindenter manufacturers (e.g., Hysitron and Zwick) use PC as a reference for their nanoindentation systems for testing polymers. The mean values of the complex moduli of 20 dynamic microindentations of the unannealed and the annealed samples were (3217 ± 729) MPa and (2402 ± 253) MPa, respectively. The result of the unannealed sample obviously shows that stresses caused by processing has an immense influence on the outcome compared to the annealed sample, where these processing stresses were (partially) relaxed during annealing. The standard deviation of both mean values also indicates that the effect of these processing stresses can locally differ enormously.

Moreover, the complex modulus of HDPE determined with the cone indenter, as is shown in Table 4, shows the lowest value compared to the other indenters. To obtain a better insight into this result, unannealed and annealed (2 h at 90 °C) injection molded HDPE samples were examined both in the “shoulders” of the tensile bar (see the scheme in Figure 1), accounting for near to and far from the gate. The results presented in Table 5 confirm the differences resulting from the processing treatment. The unannealed HDPE sample shows a lower complex modulus for the far from the gate region as for the near to the gate region. The far from the gate region experiences faster cooling rates during processing, which causes a lower degree of crystallinity, and therefore the lower complex modulus. In contrast, the near to the gate region experiences lower cooling rates due to the fact that the gate needs to remain open at higher temperatures for the holding pressure phase. These lower cooling rates cause higher crystallinities, which in turn cause the higher complex moduli. During annealing at elevated temperature, the amorphous areas are partially able to crystallize due to a higher mobility of macromolecules, however limited by polymer entanglements. This crystallization effect is more distinct for the samples far from the gate. Near the gate, the effect of annealing is hardly detectable since this area experiences a slower cooling during injection molding.

Another highlight of the development of this microindentation system is the possibility to spatially resolve dynamic microindentations. This is displayed on the cross-section surface of the narrow parallel area in the middle of the unannealed injection molded HDPE tensile bar. A 20 mm long sample was mounted with a cold curing two-component embedding epoxy compound (SpeciFix 20, Struers, Willich, Germany). After a complete curing, a 5 mm thick plane-parallel slice of the embedded sample was cut with an annular saw (SP1600, Leica, Wetzlar, Germany) under steady water cooling. The sample was then placed on the sample holder of the x-y-stage between the rectangular angled hooks and fixed into place by the tension spring. With the x-y-positioning lasers, the indenter was positioned 200 µm from the edge above the sample. A position nearer to the edge of the embedding material would lead to false results caused by the stiffening effect of the embedding material during dynamic microindentation. Across the cross-section surface, measurements were performed every 400 µm (three-fold determination), taking into account at least two times indentation–diameter–distance (2*d*) between two indentations. 2*d* between indentations is needed to avoid material deformation from previous indentations influencing subsequent indentations.

**Figure 5:**
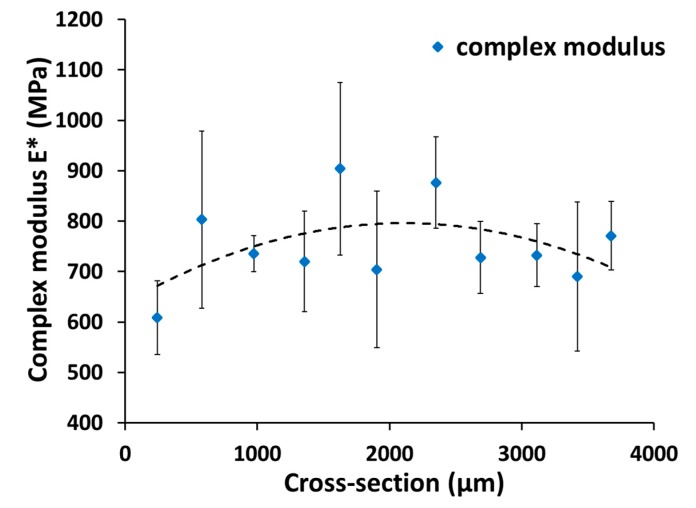
Complex modulus across the cross-section of unannealed HDPE; second order polynomial fit as a trend (dotted) line.

The results of the spatially resolved microindentations across the cross-section surface of the unannealed HDPE tensile bar are shown in Figure 5. Second order polynomial fit is displayed as a trend line to show the course of the results across the cross-section surface. Close to the edges of the sample, near 0 and 4000 µm, the complex moduli show lower values than in the middle of the cross-section. Due to the fast cooling rates close to the mold during injection molding, fewer crystalline regions were formed near the edges and, therefore, the lower complex moduli were measured. However, in the center of the cross-section, the cooling rates were slower, and thus, more crystalline regions were formed, leading to the higher complex moduli.

Overall, it can be seen that the sensitivity and the reproducibility of the dynamic microindentation with a tungsten cone indenter are good enough to distinguish between small local differences in mechanical properties, and the specially developed x-y-stage enables the spatial resolution of these mechanical properties.

## 5. Conclusions

This paper reflects some of the issues related to the tungsten cone indenters of a newly developed [5] quasi-static and dynamic microindentation system by implementing diamond and tungsten cone indenters into a conventional DMA. Dynamic microindentations were performed on high density polyethylene (HDPE) with three different tungsten welding electrodes (1 mm diameter) as a cone indenter to obtain optimal measuring conditions. In addition, dynamic microindentations were performed on polybutylene terephthalate, polycarbonate, and thermoplastic polyurethane. The results showed that for the tungsten electrode with a 2% lanthanum cone indenter, the first order of the resonant frequency was less than 10%, which corresponds to the manufacturer’s specifications. Furthermore, this cone indenter showed a sinusoidal raw response signal, and its geometry remained unaffected after 250 indentations.

The proposed microindentation system is able to intercept and distinguish among various processing influences as well as heat treatment-induced inhomogeneities. A comparison of the complex moduli measured by dynamic microindentation and the values from dynamic microindentations with diamond indenters from the previous study, three-point bending, and literature reveals a good agreement. Additionally, dynamic microindentations on the cross-section surface of the unannealed HDPE tensile bar with the specially developed x-y-stage showed that small local differences in mechanical properties can be spatially resolved.

This study demonstrated that dynamic microindentation with a tungsten cone indenter implemented into a conventional DMA is an adequate method to determine spatially resolved local viscoelastic surface properties. This enhances and finalizes the microindentation system which allows spatially resolved local as well as bulk viscoelastic material information to be obtained within one instrument.

## Figures and Tables

**Table 1:** Investigated polymers.

Polymer	Standard	Young’s modulus ^1^ (MPa)	Young’s modulus range [27,28] (MPa)	Poisson’s ratio [29]
PBT (Vestodur, Evonik, Essen, Germany)	Injection molded tensile test bar ISO 527-2: 1A	2600 (ISO 527-1/-2)	2500–2800	0.42
PC (Makrolon, Bayer, Leverkusen, Germany)	Injection molded tensile test bar ISO 527-2: 1A	2400 (ISO 527-1/-2)	2200–2600	0.41
HDPE (Lupolen, LyondellBasell, Wesseling, Germany)	Injection molded tensile test bar ISO 527-2: 1A	900 (ISO 527-1/-2)	600–1400	0.46
TPU (Desmopan, Covestro, Leverkusen, Germany)	n.a.	29 (ISO 6721-1/-4)	20–400	0.49

^1^ Values according to the material datasheet.

**Table 2:** Investigated tungsten welding electrodes as cone indenters.

Cone indenter	Diameter (mm)	Tip angle (°)	Tip radius (µm)	Abbreviation
Tungsten electrode, W > 99.9%	1.0	28	8	Green
Tungsten electrode + 2% lanthanum	1.0	28	5	Blue
Tungsten electrode + rare earth elements	1.0	28	5–10	Lymox

**Table 3:** Overtones of the first order resonant frequencies of various tungsten cone indenters and complex moduli of high-density polyethylene (HDPE) measured under different loads.

	Green Indenter		Lymox Indenter		Blue Indenter	
Load (N)	Overtones (%)	E* (MPa)	Overtones (%)	E* (MPa)	Overtones (%)	E* (MPa)
0.5	7.5 ± 0.8	1732 ± 167	6.6 ± 3.1	3487 ± 335	4.0 ± 0.6	1780 ± 162
0.75	8.4 ± 1.9	1565 ± 243	6.8 ± 2.0	1843 ± 409	5.3 ± 1.8	1643 ± 126
1.0	8.4 ± 1.0	1652 ± 151	8.8 ± 2.0	1387 ± 237	5.7 ± 1.1	1343 ± 428
1.25	6.8 ± 0.9	1082 ± 114	8.7 ± 1.1	1117 ± 92	5.0 ± 2.3	1119 ± 88

**Table 4:** Complex moduli for investigated polymers obtained with various indenters, three-point bending, and Young’s moduli from literature [27,28].

Polymer	Tungsten cone indenter (MPa)	Vickers diamond indenter [5] (MPa)	Berkovich diamond indenter [5] (MPa)	Rockwell diamond indenter [5] (MPa)	DMA three-point bending [5] (MPa)	Young’s modulus literature [27,28] (MPa)
PBT	2890 ± 378	1919 ± 353	1737 ± 358	1821 ± 78	2787 ± 84	2500–2800
PC	2726 ± 386	2289 ± 156	1957 ± 317	2142 ± 351	2380 ± 19	2200–2600
HDPE	1053 ± 175	1381 ± 339	1929 ± 302	1553 ± 75	1565 ± 33	600–1500
TPU	38 ± 2	24 ± 2	26 ± 3	24 ± 3	73 ± 3	20–400

**Table 5:** Complex moduli (MPa) of unannealed and annealed HDPE for gate far and gate near.

HDPE	Gate far	Gate near
Unannealed	791 ± 132	1095 ± 76
Annealed	1073 ± 231	1073 ± 156
